# Stratification of Early Arrhythmic Risk in Patients Admitted for Acute Coronary Syndrome: The Role of the Machine Learning‐Derived “PRAISE Score”

**DOI:** 10.1002/clc.70035

**Published:** 2024-12-19

**Authors:** Luca Cumitini, Ailia Giubertoni, Lidia Rossi, Domenico D'Amario, Leonardo Grisafi, Paola Abbiati, Fabrizio D'Ascenzo, Gaetano Maria De Ferrari, Giuseppe Patti

**Affiliations:** ^1^ Department of Translational Medicine University of Eastern Piedmont Novara Italy; ^2^ Division of Cardiology Maggiore della Carità Hospital Novara Italy; ^3^ Cardiovascular and Thoracic Department Division of Cardiology, “Città della Salute e della Scienza” Hospital Turin Italy; ^4^ Department of Medical Sciences University of Turin Turin Italy

**Keywords:** acute coronary syndrome, arrhythmias, machine learning, PRAISE risk score

## Abstract

**Background:**

The PRAISE (PRedicting with Artificial Intelligence riSk aftEr acute coronary syndrome) score is a machine learning‐based model for predicting 1‐year adverse cardiovascular or bleeding events in patients with acute coronary syndrome (ACS). Its role in predicting arrhythmic complications in ACS remains unknown.

**Methods:**

Atrial fibrillation (AF) and ventricular arrhythmias (VA) were recorded by continuous electrocardiographic monitoring until discharge in a cohort of 365 participants with ACS prospectively enrolled. We considered two separate timeframes for VA occurrence: ≤ 48 and > 48 h. The objective was to evaluate the ability of the PRAISE score to identify ACS patients at higher risk of in‐hospital arrhythmic complications.

**Results:**

ROC curve analysis indicated a significant association between PRAISE score and risk of both AF (AUC 0.89, *p* = 0.0001; optimal cut‐off 5.77%) and VA (AUC 0.69, *p* = 0.0001; optimal cut‐off 2.17%). Based on these thresholds, high/low AF PRAISE score groups and high/low VA PRAISE score groups were created, respectively. Patients with a high AF PRAISE score more frequently developed in‐hospital AF (19% vs. 1%). Multivariate analysis showed a high AF PRAISE score risk as an independent predictor of AF (HR 4.30, *p* = 0.016). Patients with high VA PRAISE scores more frequently developed in‐hospital VA (25% vs. 8% for VA ≤ 48 h; 33% vs. 3% for VA > 48 h). Multivariate analysis demonstrated a high VA PRAISE score risk as an independent predictor of both VA ≤ 48 h (HR 2.48, *p* = 0.032) and VA > 48 h (HR 4.93, *p* = 0.014).

**Conclusion:**

The PRAISE score has a comprehensive ability to identify with high specificity those patients at risk for arrhythmic events during hospitalization for ACS.

## Graphical abstract

PRAISE score is a machine learning‐derived tool with a comprehensive capability to predict arrhythmic complications. The score is easy to use and performs well, and a high score can identify with great specificity those patients at risk for ventricular and atrial arrhythmias during hospitalization. ACS, acute coronary syndrome; AF, atrial fibrillation; AUC, area under the curve; h, hours; PCI, percutaneous coronary intervention; PRAISE, PRedicting with Artificial Intelligence riSk aftEr acute coronary syndrome; VA, ventricular arrhythmias

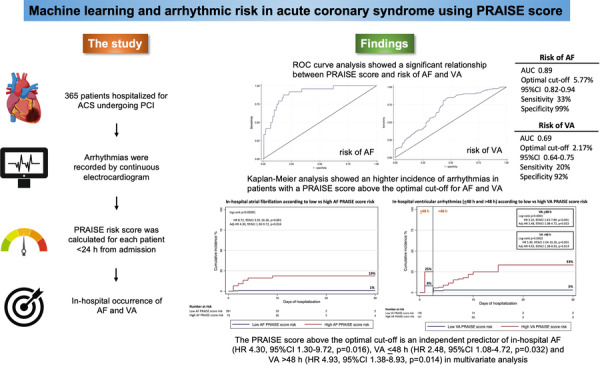

**Abbreviations**
ACE: angiotensin‐converting enzyme
ACS: acute coronary syndrome
AF: atrial fibrillation
ARB: angiotensin receptor blockers
AUC: area under the curve
BARC: Bleeding Academic Research Consortium
BMI: body mass index
CABG: coronary artery bypass graft
CI: confidence interval
EKG: electrocardiogram
HR: hazard ratio
LVEF: left ventricular ejection fraction
MI: myocardial infarction
NSTEMI: non‐ST‐segment elevation myocardial infarction
NSVT: nonsustained ventricular tachycardia
PCI: percutaneous coronary intervention
PRAISE: predicting with artificial intelligence risk after acute coronary syndrome
ROC: receiver operating characteristic
STEMI: ST‐segment elevation myocardial infarction
SVT: sustained ventricular tachycardia
UA: unstable angina
VA: ventricular arrhythmias
VT: ventricular tachycardia

Atrial and ventricular arrhythmias (VA) may occur in the setting of patients with acute coronary syndromes (ACS). In particular, here, atrial fibrillation (AF) is frequent, with an incidence ranging from 5% to 23%, and is associated with lower survival and worse clinical outcomes, mainly due to cardioembolic complications and development of heart failure, as compared with patients maintaining a normal sinus rhythm [1, 2]. The course of ACS patients may be also complicated by sustained ventricular tachyarrhythmias, such as ventricular tachycardia (VT) or ventricular fibrillation (VF), caused by ongoing myocardial ischemia, elevated sympathetic tone, development of pro‐arrhythmic myocardial scar tissue, increase in circulating catecholamines or electrolyte disturbances [3, 4]. The occurrence of early VA in ACS may increase in‐hospital mortality.

Based on the above, the stratification of ACS patients for the risk of early developing VA or AF carries important prognostic and therapeutic implications [5, 6]. The PRAISE (PRedicting with Artificial Intelligence riSk aftEr acute coronary syndrome) score [7] was recently designed to develop a machine learning‐based risk stratification model able to predict the risk of all‐cause death, recurrent myocardial infarction (MI) and major bleeding after ACS and guide clinical decision making. To date, its role in predicting arrhythmic complications in ACS is unknown.

The aim of this prospective study was to evaluate the potential role of the PRAISE score in stratifying at baseline ACS patients undergoing percutaneous coronary intervention (PCI) according to the risk of VA and AF during a hospital stay.

## 1 Methods

### 1.1 Study Population and Data Collection

This is a prospective, observational study performed at the Cardiology Unit of Maggiore della Carità Hospital in Novara, Italy. Consecutive patients hospitalized for ACS (unstable angina [UA], non‐ST‐segment elevation myocardial infarction [NSTEMI], or ST‐segment elevation myocardial infarction [STEMI]) and receiving PCI from October 2021 to January 2023 were enrolled. Exclusion criteria were: a conservative strategy for the index event (e.g., no invasive approach with coronary angiography); revascularization with coronary artery bypass graft (CABG) after coronary angiography; prior history of AF; left ventricular ejection fraction (LVEF) < 15%. Individual data, including demographic and physical characteristics, medical history, cardiovascular risk factors, laboratory and echocardiographic findings, medical therapy, angiographic/procedural features, and clinical events from admission to discharge were collected. The PRAISE risk score was calculated for each patient < 24 h from admission.

### 1.2 Ethics

The study was designed following Declaration of Helsinki, Good Clinical Practice guidelines and other related guiding principles. According to the design, all results were used only for scientific purposes, without uncovering personal identifiers. The authors had full access to all data and take responsibility for their integrity and analyses. The protocol was approved by local Ethic Committee.

### 1.3 PRAISE Data Set and Classes of Risk

In detail, the PRAISE data set [7, 8] consisted of a derivation cohort of 19 826 patients with ACS and 1‐year follow‐up from two registries (BleeMACS [Bleeding complications in a Multicenter registry of patients discharged after an Acute Coronary Syndrome] and RENAMI [REgistry of New Antiplatelets in patients with Myocardial Infarction]) and an external validation cohort of 3444 patients with ACS and 2‐year follow‐up from three collected sources (SECURITY [Second Generation Drug‐Eluting Stent Implantation Followed by 6‐ vs. 12‐month Dual Antiplatelet Therapy] randomized trial, FRASER [frailty in elderly patients receiving cardiac interventional procedures] registry, and ARYOSTO [Prospective Registry of Acute Coronary Syndromes in Ferrara] registry). The structured data set included 25 different variables: 16 clinical variables (age, gender, diabetes mellitus, systemic hypertension, hyperlipidemia, peripheral artery disease, estimated glomerular filtration rate [modification of diet in renal disease formula], prior MI, prior PCI, CABG, prior stroke, prior bleeding, malignancy, STEMI presentation, hemoglobin, and LVEF); 5 therapeutic variables (beta‐blocker, angiotensin‐converting enzyme inhibitor/angiotensin‐receptor blocker, statin, oral anticoagulation, and proton pump inhibitor); 2 angiographic variables (multivessel disease and complete revascularization); 2 procedural variables (vascular access and PCI with drug‐eluting stent). The PRAISE score was able to separately predict the occurrence of three different outcomes 1 year after discharge: all‐cause death, new event of MI and major bleeding (by 3 or 5 Bleeding Academic Research Consortium [BARC] definition) [9]. Patients from the pooled study cohorts were divided for each outcome measure into estimated risk deciles and then grouped into levels of low, moderate and high risk with thresholds reflecting clinically meaningful gradients in risk from one group to the next. Such stratification is meant to highlight the clinical implication of each risk value computed by the model. The calculator of the score is available online at https://praise.hpc4ai.it/.

### 1.4 Study Endpoints

Study endpoints were in‐hospital incidence of new‐onset AF and VA. As early VA occurring during the first 2–3 days after the clinical presentation are usually related to dynamic ischemia and/or reperfusion and are not considered a marker of future clinical risk [4], we have decided to explore separately two timeframes of VA occurrence: ≤ 48 and > 48 h. In this regard, patients were monitored during the cardiac care unit and ward stay by continuous standard 12‐lead 24‐h electrocardiogram (EKG) until discharge. A system for centralized presentation of EKG was used (Dynascope DS‐8500; Fukuda Deshi). One member of the staff (one nurse) was always present for monitoring the occurrence of arrhythmias. Every hour all arrhythmias were systematically classified and reported in the data set.

For the study endpoints, the following definitions were utilized:

- AF: at least one episode of supraventricular tachyarrhythmia with uncoordinated atrial electrical activation and irregular R–R intervals, absence of distinct repeating P waves and irregular atrial activations lasting at least 5 min.
- VA: at least one episode of non‐sustained VT (NSVT), defined as three or more consecutive ventricular complexes at a rate > 100 beats/min; sustained VT (SVT), defined as ventricular rhythm faster than 100 beats/min typically lasting at least 30 s or requiring termination earlier due to hemodynamic instability; VF, defined as a chaotic rhythm with undulations that are irregular in timing and morphology, without discrete QRS complexes on the surface EKG.

For the purpose of the study, among the three different PRAISE scores (PRAISE score for all‐cause death; PRAISE score for MI; PRAISE score for major bleeding), we used for full analyses that score having in our population the highest area under the curve (AUC) for in‐hospital VA and AF. In particular, AUC derived from the PRAISE score regarding all‐cause death was 0.89 for AF and 0.69 for VA, regarding MI was 0.87 for AF and 0.65 for VA, and regarding major bleeding was 0.82 for AF and 0.65 for VA. Thus, due to its highest AUC value, in the present investigation we explored the predictive role of PRAISE score for the occurrence of VA and AF by using the score for all‐cause mortality.

### 1.5 Statistical Analysis

For continuous variables, data are reported as median and quartile 1 to quartile 3 (interquartile range) for variables with skewed distributions or mean and standard deviation for variables with normal distribution. The presence of normal distribution was verified by Shapiro–Wilks test. Categorical variables are indicated as absolute numbers and percentages. For statistical comparison, the Mann–Whitney Test was used for continuous non‐normal variables and the Student's *t*‐test for normal variables. Chi‐square or Fisher Exact test was used for categorical variables, where appropriate. The area under the receiver operating characteristic curve (ROC) was computed to evaluate the discriminative ability of the prediction of the PRAISE score for in‐hospital arrhythmias. To determine the optimal cut‐off value having maximal sensitivity and specificity, we utilized Youden's index. Therefore, patients were divided into high and low AF PRAISE score groups and into high and low VA PRAISE score groups, according to the aforementioned threshold values. In‐hospital arrhythmia rates were estimated by the Kaplan–Meier method and presented as incidence rate curves in patients with high versus low AF PRAISE score risk and with high versus low VA PRAISE score risk. In Kaplan–Meier analysis, the comparison between the two incidence rate curves was performed by the log‐rank test. The Cox proportional hazards model was used to estimate the independent association between high AF/VA PRAISE score risk at baseline and in‐hospital arrhythmias. All parameters reported in Table 1 were included in the Cox model, where a stepwise approach was utilized to identify independent predictors. In particular, variables with a *p* value < 0.05 at univariate analysis were then entered into the multivariate analysis. Hazard ratio (HR) and 95% confidence interval (CI) were then calculated. Statistical significance was indicated by a significance level of *p* value < 0.05. Analysis of data and generation of statistical models were performed using the statistical software STATA 18.0 (StataCorp, LP, College Station, TX, USA).

**Table 1:** Baseline features of the population.

Overall population (*n* = 365)	
Age (years)	67 (57–75)
Gender	
Men	283 (78)
Women	82 (22)
BMI (Kg/m^2^)	26 (23.8–29.1)
Systemic hypertension	202 (55)
Dyslipidemia	130 (35)
Diabetes mellitus	50 (14)
Chronic renal failure	73 (20)
Previous cerebral ischemic event	13 (4)
Peripheral vascular disease	26 (7)
Previous myocardial infarction	56 (15)
Clinical presentation	
STEMI	254 (70)
NSTEMI	106 (29)
Unstable angina	5 (1)
LVEF (%)	49 (42–55)
Mitral regurgitation (moderate to severe)	41 (11)
Hemoglobin (g/dL)	14 (12.6–15.2)
eGFR (mL/min)	89 (64–100)
Diseased coronary vessels	
1	154 (42)
2	129 (35)
3	82 (23)
Multivessel PCI	210 (58)
In‐hospital therapy	
Aspirin	365 (100)
Clopidogrel	43 (12)
Ticagrelor	285 (78)
Prasugrel	37 (10)
Beta‐blocker	337 (92)
ACE inhibitors/ARB	314 (86)
Statin	361 (99)

*Note:* Values are given as median (interquartile range) or *n* (%).

Abbreviations: ACE, angiotensin‐converting enzyme; ARB, angiotensin receptor blockers; BMI, body mass index; eGFR, estimated glomerular filtration rate; LVEF, left ventricular ejection fraction; NSTEMI, non‐ST‐elevation myocardial infarction; PCI, percutaneous coronary intervention; STEMI, ST‐segment elevation myocardial infarction.

## 2 Results

**Figure 1:**
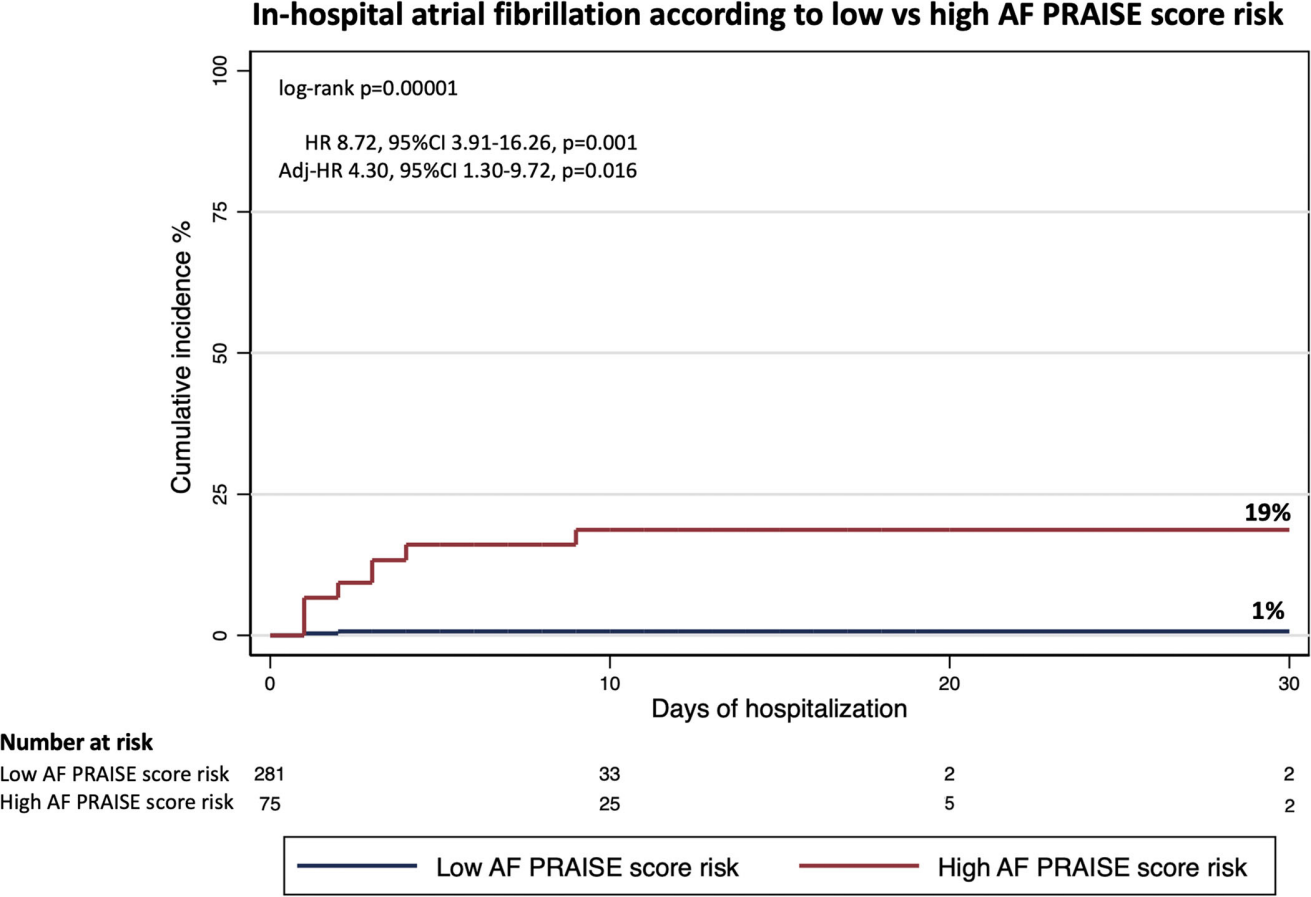
Kaplan–Meier curves for incidence of in‐hospital atrial fibrillation in patients with high versus low AF PRAISE score risk. AF, Atrial fibrillation; PRAISE, PRedicting with Artificial Intelligence riSk aftEr acute coronary syndrome.

A total of 365 patients were enrolled in the study. The flowchart leading to the final sample size is indicated in Supporting Information S1: Figure 1. The main characteristics of the population are reported in Table 1. Most participants were hospitalized for STEMI (70%, *n* = 254), with 29% having NSTEMI (*n* = 106) and 1% UA (*n* = 5). During the hospital stay, 36% of patients (*n* = 133) had at least one episode of VA, and 7% (*n* = 24) had at least one episode of new‐onset AF (Supporting Information S4: Table 1). Specifically, 36% of patients (*n* = 131) had at least one episode of NSVT, 2% (*n* = 9) had at least one episode of SVT, and 2% (*n* = 6) had at least one episode of VF. In‐hospital mortality was 1% (*n* = 3).

### 2.1 In‐Hospital AF

**Figure 2:**
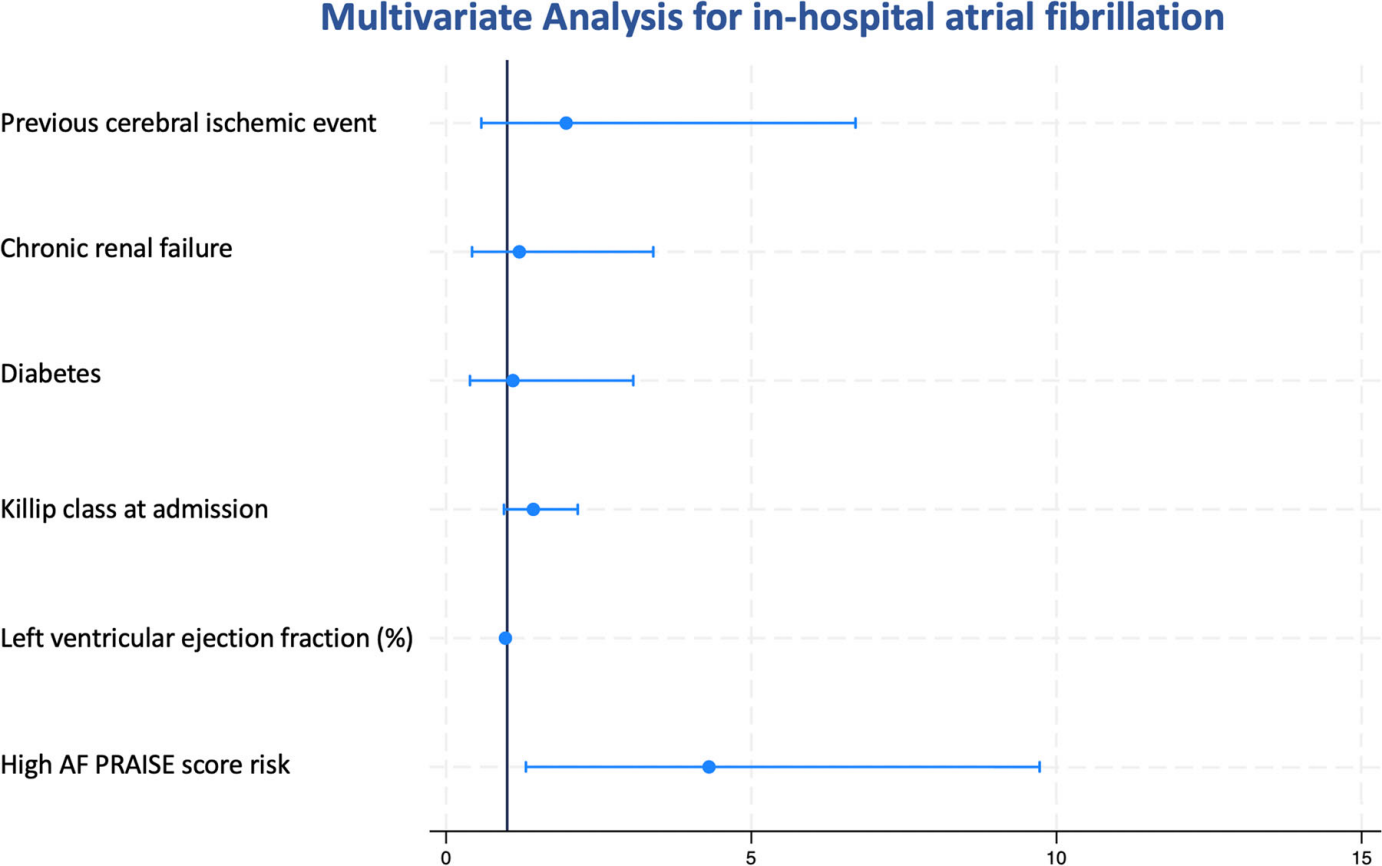
Multivariate analysis for in‐hospital atrial fibrillation. AF, atrial fibrillation; PRAISE, PRedicting with Artificial Intelligence riSk aftEr acute coronary syndrome.

ROC curve analysis (Supporting Information S2: Figure 2) indicated a significant relationship between the PRAISE score and risk of AF during hospital stay (AUC 0.89, 95% CI 0.82–0.94, *p* = 0.0001). The PRAISE score risk had a sensitivity of 33% and a specificity of 99% for in‐hospital AF. The optimal cut‐off value for the PRAISE score risk to predict the occurrence of in‐hospital AF was 5.77%. We categorized the participants according to such threshold value, assigning 282 patients to the low AF PRAISE score risk group and 83 patients to the high AF PRAISE score risk group. Figure 1 illustrates Kaplan–Meier curves for in‐hospital AF in patients with high versus low AF PRAISE score risk, with a cumulative incidence of 19% versus 1%, respectively (log‐rank *p* = 0.00001). Multivariate analysis showed that a high AF PRAISE score risk was an independent predictor of in‐hospital AF (HR 4.30, 95% CI 1.30–9.72, *p* = 0.016) (Figure 2). A previous cerebral ischemic event (HR 1.96, 95% CI 0.57–6.70, *p* = 0.042) also increased the risk of developing AF. For the relationship between PRAISE score and risk of AF, no difference between patients with UA/NSTEMI versus STEMI patients was found (*p* = 0.26).

### 2.2 In‐Hospital VA

**Figure 3:**
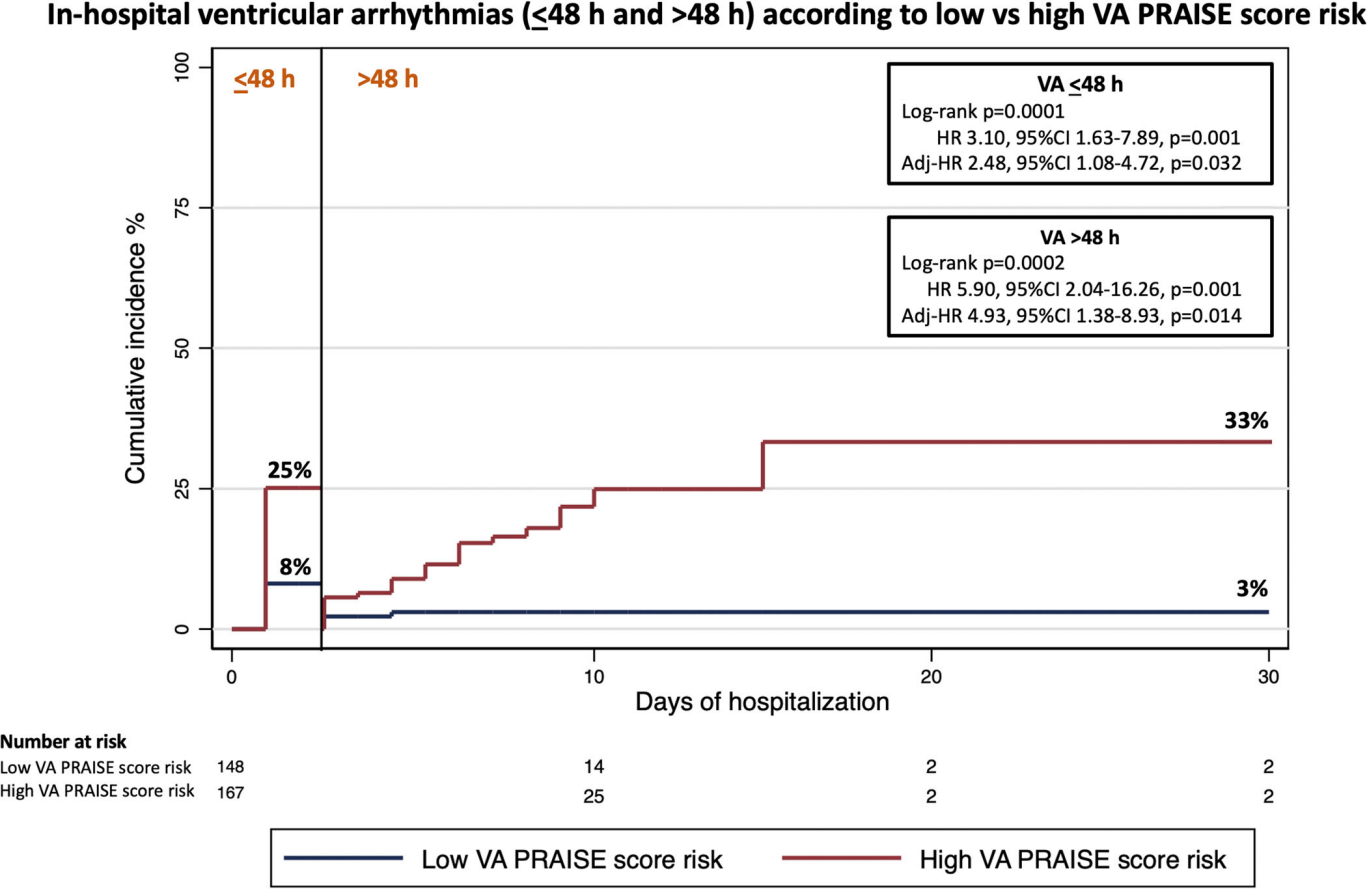
Kaplan–Meier curves for incidence of in‐hospital ventricular arrhythmias (≤ 48 and > 48 h) in patients with high versus low VA PRAISE score risk. PRAISE, PRedicting with Artificial Intelligence riSk aftEr acute coronary syndrome; VA, ventricular arrhythmias.

**Figure 4:**
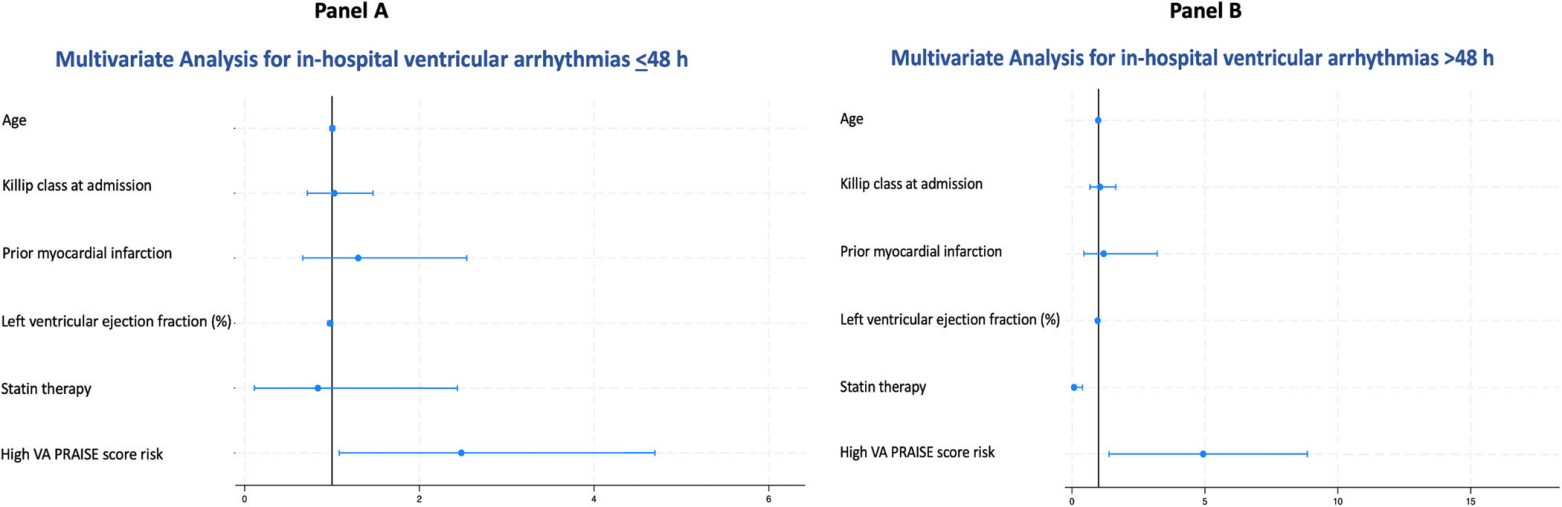
Multivariate analysis for in‐hospital ventricular arrhythmias ≤ 48 h (panel A) and > 48 h (panel B). PRAISE, PRedicting with Artificial Intelligence riSk aftEr acute coronary syndrome; VA, ventricular arrhythmias.

ROC curve analysis (Supporting Information S3: Figure 3) showed a significant predictive value of the PRAISE score for developing VA (AUC 0.69, 95% CI 0.64–0.75, *p* = 0.0001) during hospital stay. The PRAISE score risk had a sensitivity of 20% and a specificity of 92% for in‐hospital VA. The optimal cut‐off value for the PRAISE score risk to predict the occurrence of in‐hospital VA was 2.17%. Thus, we categorized the participants according to such threshold value, assigning 159 patients to the low VA PRAISE score risk group and 206 patients to the high VA PRAISE score risk group. Figure 3 shows Kaplan–Meier curves for in‐hospital VA in patients with high versus low VA PRAISE score risk, with a cumulative incidence of 25% versus 8%, respectively, for in‐hospital VA ≤ 48 h (log‐rank *p* = 0.0001) and of 33% versus 3%, respectively, for in‐hospital VA > 48 h (log‐rank *p* = 0.0002). Multivariate analysis demonstrated that a high VA PRAISE score risk was an independent predictor of both in‐hospital VA ≤ 48 h (HR 2.48, 95% CI 1.08–4.72, *p* = 0.032) and in‐hospital VA > 48 h (HR 4.93, 95% CI 1.38–8.93, *p* = 0.014) (Figure 4). A preserved LVEF (HR 0.96, 95% CI 0.92–1.00, *p* = 0.045) and the use of statin therapy (HR 0.07, 95% CI 0.01–0.38, *p* = 0.034) were associated with a significant lower risk of in‐hospital VA > 48 h. For the relationship between PRAISE score and risk of VA, no difference between patients with UA/NSTEMI versus STEMI patients was found (*p* = 0.39).

## 3 Discussion

Machine learning algorithms, exploring high‐dimensional and nonlinear relations among features, could represent a novel approach to the compelling requirement of a personalized risk assessment. Indeed, the PRAISE score, derived from a machine learning process, in the derivation and validation cohorts appeared to overperform for the stratification of the risk of adverse events compared to existing ischemic and bleeding risk scores [7, 10]. Notably, in a recent work we found that the adoption of the PRAISE score was effective for addressing the issue of a tailored intensity of dual antiplatelet therapy after ACS based on patients' offsetting bleeding and ischemic profiles [8]. To our knowledge, the present study first investigated the potential role of the PRAISE score in predicting the development of in‐hospital cardiac arrhythmias in patients hospitalized for ACS, the large majority for MI.

AF development is associated with increased morbidity and mortality in patients with ACS. This association, to some extent, is a function of concomitant pathological conditions, since “lone” AF in younger patients without structural heart disease is not a predictor of poorer outcome and higher mortality [11]. Nonetheless, in patients with acute MI, new‐onset AF is documented as an independent and powerful prognostic factor for adverse events. In the OPTIMAAL trial [12], the development of new‐onset AF was related to a 3.8‐ and 1.8‐fold increase in mortality at 30 days and over the entire study course, respectively. In our cohort, the incidence of AF was 7%, similar to what was observed in previous large epidemiological studies [13]. We found a significant direct relationship between PRAISE score and risk of in‐hospital AF: patients with a PRAISE score above the cut‐off of 5.77% at baseline were more likely to develop AF than those with a score below this threshold (19% vs. 1%). Moreover, the specificity of the PRAISE score for developing AF was very high (99%), and a high AF PRAISE score was associated with a 4.3‐fold increased risk of AF at multivariate analysis. Another independent predictor was a previous cerebral ischemic event. Of note, patients with a high AF PRAISE score had a more elevated prevalence of previous cerebral ischemic events, possibly due to occult AF episodes.

Patients with VA complicating an acute MI present a higher mortality, especially when arrhythmic episodes are expression of depressed left ventricular function [14]. There is a temporal distribution of VA in patients with acute MI: early VA occurs up to 48–72 h, for example, during a period characterized by dynamic ischemia and reperfusion, whereas VA from 72 h to the first weeks postevent reflects a more chronic phase when cardiac remodeling continues to occur. A prompt revascularization with PCI and the use of beta‐blockers, while resulting in modification of the natural history of the disease, reduce the incidence of VT or FV occurring < 48 h from acute MI [15]. However, the relationship between early VA and subsequent mortality remains controversial. Conversely, patients who develop sustained VF or VT > 48 h from the index MI have an increased risk of death during follow‐up, often as a consequence of heart failure with reduced ejection fraction. Therefore, we explored separately the two timeframes of VA occurrence: ≤ 48 and > 48 h. Notably, prevention of VA in the postacute phase of MI remains an area of active investigation, with data suggesting that the use of antiplatelet agents, angiotensin‐converting enzyme inhibitors, or statins may reduce the subsequent arrhythmic risk [16, 17, 18]. Nevertheless, approximately 10% of post‐MI survivors are considered at increased risk of dying in the first months or years after discharge [19], and sudden death due to sustained VA accounts for approximately 50% of death causes in these high‐risk patients [20].

In the present study, more than one‐third of patients had sustained VA, occurring mainly within 2 weeks from admission. We observed a significant relationship between the PRAISE score and risk of VA. In particular, patients with a high VA PRAISE score at baseline (> 2.17%) were more likely to develop VA during hospitalization compared to those with a low score, and this occurred for both VA ≤ 48 h (25% vs. 8%) and VA > 48 h (33% vs. 3%). Notably, the specificity of the PRAISE score for developing VA was 92% and a high VA PRAISE score was associated with approximately a 2.5‐fold increased risk of VA ≤ 48 h and a 4.9‐fold increased risk of VA > 48 h at multivariate analysis. Notably, independent predictors of reduced arrhythmic risk for VA > 48 h were preserved LVEF and statin use. Various pleiotropic mechanisms for which statins may protect against the development of VA have been proposed, including potential membrane‐stabilizing properties, improvement in autonomic function and anti‐inflammatory properties [21]. In patients with acute MI, a contributing mechanism of arrhythmia reduction with statin therapy is plaque stabilization, which consequently reduces the burden of ischemic tachyarrhythmias [21, 22].

Regarding the performance of the PRAISE score for arrhythmias, our findings indicate a very high probability of developing subsequently during the hospitalization of both ventricular and atrial arrhythmias if a higher score is recorded at baseline upon admission. Conversely, the sensitivity of the PRAISE score was low, suggesting that the presence of a nonhigher score does not exclude to develop future arrhythmic episodes over the short term. This latter finding is consistent with the multifactorial causes of arrhythmic complications in patients with recent MI, where multiple variables can be involved.

Our results should be interpreted in light of the study limitations, including possible biases inherent to all observational investigations. At multivariate analysis, the CIs are wide and close to unity for both endpoints, indicating a limited sample size; this may carry statistical uncertainty. Furthermore, we explored the occurrence of early arrhythmias, and therefore, the predictive role of the PRAISE score on arrhythmic complications during long‐term follow‐up needs to be evaluated by future investigations. Our results are hypothesis‐generating and merit further validation on larger, external cohorts.

In conclusion, in patients with ACS, the PRAISE score is a machine learning‐derived tool with a comprehensive capability to predict not only the risk of future death, recurrent MI, and bleeding complications but also of early arrhythmic complications. The score is easy to use and performs well, and a high score can identify with great specificity those patients at risk for ventricular and atrial arrhythmias during hospitalization. This may be crucial to adopt individualized strategies aimed at improving the clinical outcomes of patients admitted for acute MI. In particular, this approach allows to focus more attention on these higher‐risk patients, especially in terms of stricter postacute rhythm monitoring for early detection and treatment subclinical arrhythmias and management of conditions predisposing to arrhythmic complications, also including concomitant treatments and electrolyte imbalances.

## Author Contributions

All authors have significantly contributed to this work, and they have read and approved the manuscript.

## Conflicts of Interest

The authors declare no conflicts of interest.

## Supporting information

Supporting information. Figure 1. Flow‐chart leading to the final sample size of the study.

Supporting information. Figure 2. ROC curve analysis for PRAISE score and in‐hospital atrial fibrillation.

Supporting information. Figure 3. ROC curve analysis for PRAISE score and in‐hospital ventricular arrhythmias.

Supporting information. Table 1: Adverse outcomes during hospitalization.

Supporting information.

## Data Availability

Our research obtained informed consent from everyone who met the inclusion criteria. The data underlying this article are available in the article and its online supplementary material.
