# Supplementary figures and images for: Stratification of Early Arrhythmic Risk in Patients Admitted for Acute Coronary Syndrome: The Role of the Machine Learning‐Derived “PRAISE Score”
Source: Clin Cardiol. 2024 Dec 19;47(12):e70035. doi: 10.1002/clc.70035 (PMC11656403; doi:10.1002/clc.70035)

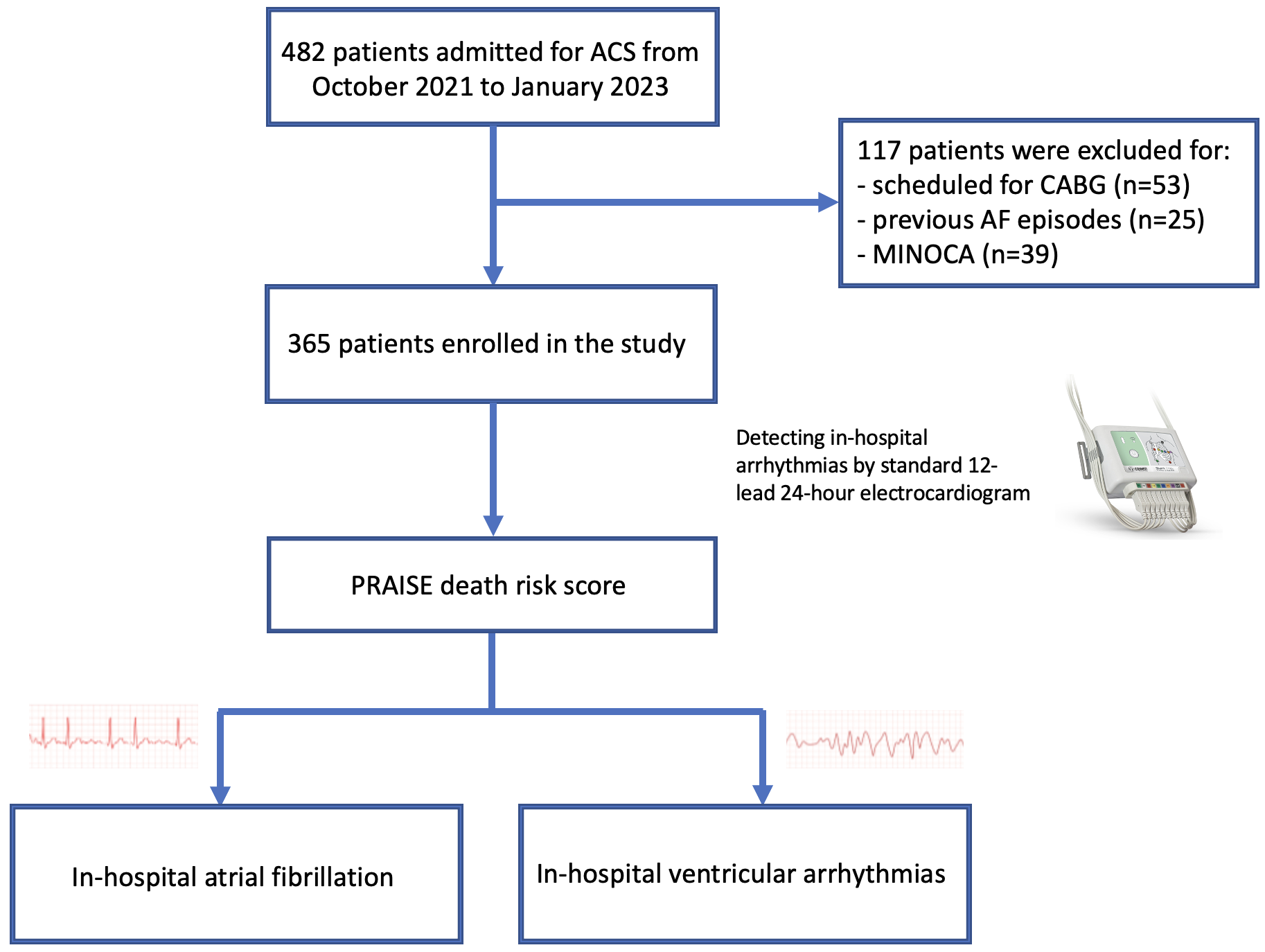

Supplement: Supplementary file 1 — Supporting information. Figure 1. Flow‐chart leading to the final sample size of the study. [file CLC-47-e70035-s002.tiff]

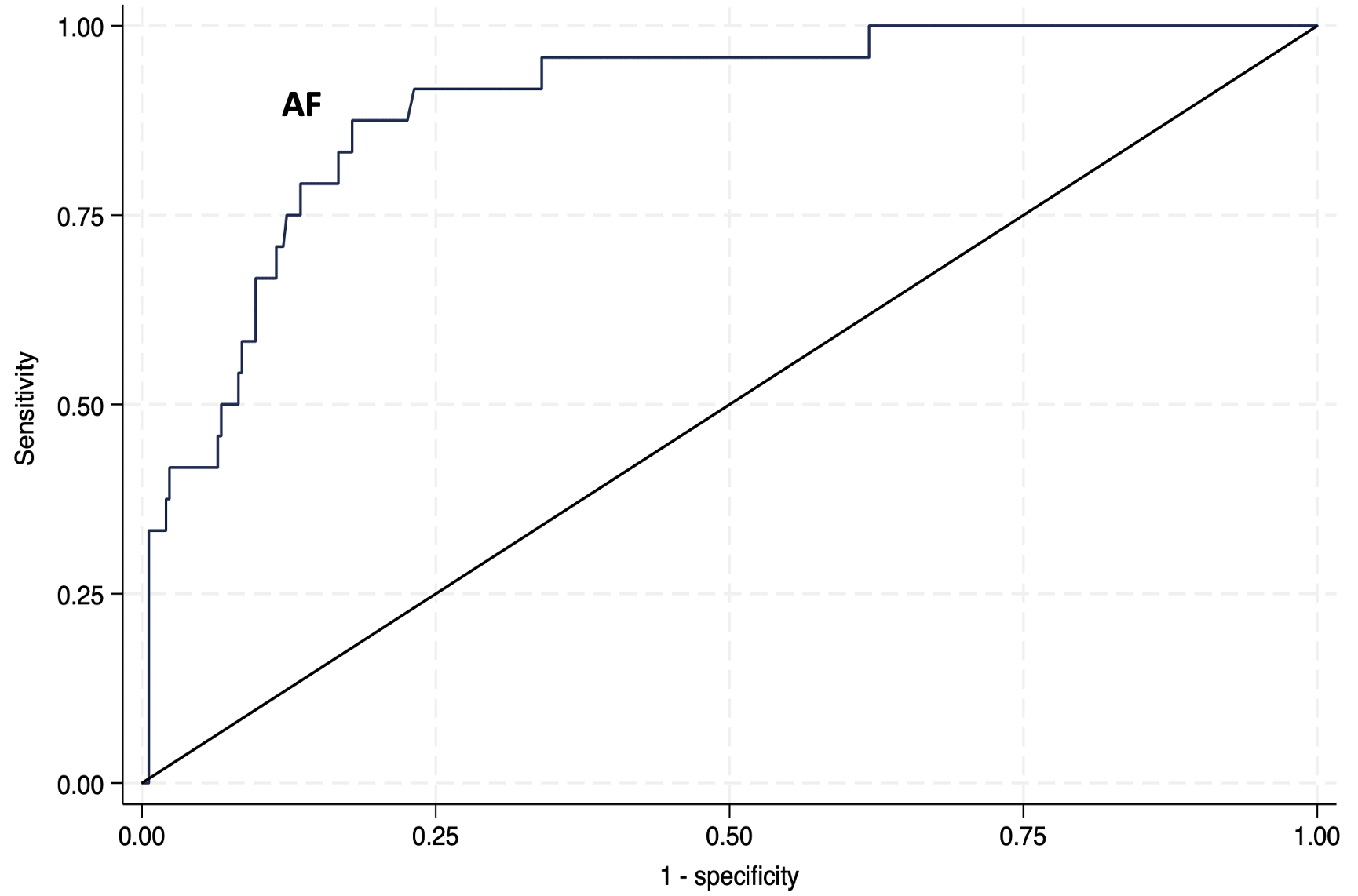

Supplement: Supplementary file 2 — Supporting information. Figure 2. ROC curve analysis for PRAISE score and in‐hospital atrial fibrillation. [file CLC-47-e70035-s001.tiff]

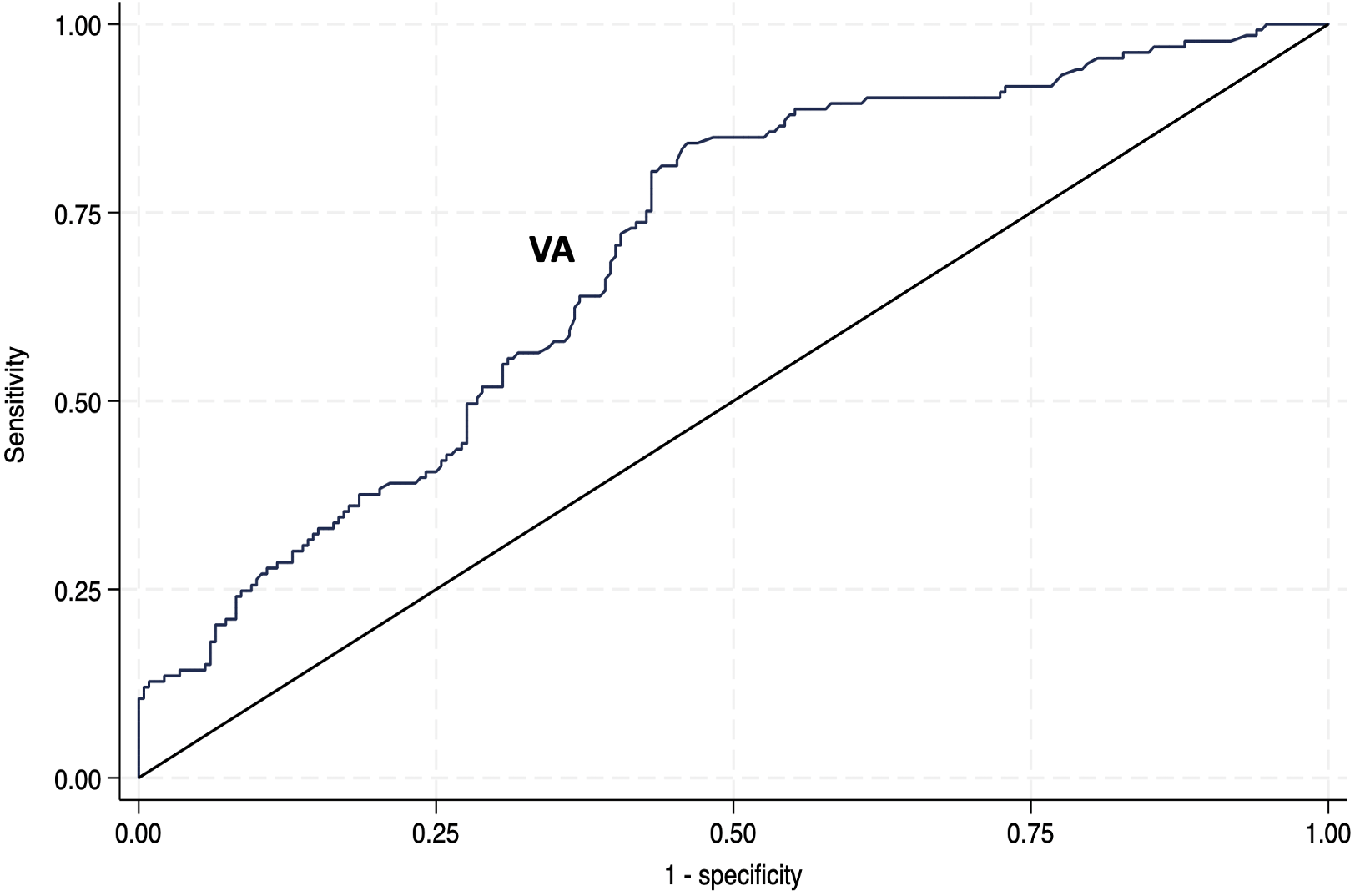

Supplement: Supplementary file 3 — Supporting information. Figure 3. ROC curve analysis for PRAISE score and in‐hospital ventricular arrhythmias. [file CLC-47-e70035-s004.tiff]
