# Supplementary material for: Stratification of Early Arrhythmic Risk in Patients Admitted for Acute Coronary Syndrome: The Role of the Machine Learning‐Derived “PRAISE Score”
Source: Clin Cardiol. 2024 Dec 19;47(12):e70035. doi: 10.1002/clc.70035 (PMC11656403; doi:10.1002/clc.70035)
Supplement: Supplementary file 4 — Supporting information. Table 1: Adverse outcomes during hospitalization. [file CLC-47-e70035-s005.docx]

| Adverse outcomes during hospitalization | |
| --- | --- |
| Atrial fibrillation | 24 (7) |
| Ventricular arrhythmias | 133 (36) |
| At least one episode of NSVT | 131 (36) |
| At least one episode of SVT | 9 (2) |
| At least one VF episodes | 6 (2) |
| In-hospital death | 3 (1) |

**Supplementary Table 1. Adverse outcomes during hospitalization**.

Values are given as n. (%).

NSVT indicates non-sustained ventricular tachycardia, SVT, sustained ventricular tachycardia, VT, ventricular tachycardia, VF, ventricular fibrillation.
