# Supplementary material for: Stratification of Early Arrhythmic Risk in Patients Admitted for Acute Coronary Syndrome: The Role of the Machine Learning‐Derived “PRAISE Score”
Source: Clin Cardiol. 2024 Dec 19;47(12):e70035. doi: 10.1002/clc.70035 (PMC11656403; doi:10.1002/clc.70035)
Supplement: Supplementary file 5 — Supporting information. [file CLC-47-e70035-s003.docx]

**Supplementary materials**

**Supplementary Table 1.** Adverse outcomes during hospitalization.

Values are given as n. (%).

NSVT indicates non-sustained ventricular tachycardia, SVT, sustained ventricular tachycardia, VT, ventricular tachycardia, VF, ventricular fibrillation.

**Supplementary Figure 1.** Flow-chart leading to the final sample size of the study.

ACS= Acute coronary syndrome; AF= Atrial fibrillation; CABG= Coronary Artery Bypass Grafting; MINOCA= Myocardial Infarction with No Obstructive Coronary Artery disease; PRAISE= PRedicting with Artificial Intelligence riSk aftEr acute coronary syndrome

**Supplementary Figure 2.** ROC curve analysis for PRAISE score and in-hospital atrial fibrillation.

AF= Atrial fibrillation

**Supplementary Figure 3**. ROC curve analysis for PRAISE score and in-hospital ventricular arrhythmias.

VA= ventricular arrhythmias
